# Evaluation of a Standardized Extract from* Morus alba* against *α*-Glucosidase Inhibitory Effect and Postprandial Antihyperglycemic in Patients with Impaired Glucose Tolerance: A Randomized Double-Blind Clinical Trial

**DOI:** 10.1155/2016/8983232

**Published:** 2016-11-16

**Authors:** Seung Hwan Hwang, Hong Mei Li, Soon Sung Lim, Zhiqiang Wang, Jae-Seung Hong, Bo Huang

**Affiliations:** ^1^Department of Food Science and Nutrition, Hallym University, 1 Hallymdeahak-gil, Chuncheon 24252, Republic of Korea; ^2^Department of Pharmacology, College of Medicine, Hallym University, 1 Hallymdeahak-gil, Chuncheon 24252, Republic of Korea; ^3^Department of Physical Education, Hallym University, 1 Hallymdeahak-gil, Chuncheon 24252, Republic of Korea; ^4^College of Food Science and Engineering, Jinzhou Medical University, Jinzhou 121001, China

## Abstract

To evaluate the antihyperglycemic effect of a standardized extract of the leaves of* Morus alba* (SEMA), the present study was designed to investigate the *α*-glucosidase inhibitory effect and acute single oral toxicity as well as evaluate blood glucose reduction in animals and in patients with impaired glucose tolerance in a randomized double-blind clinical trial. SEMA was found to inhibit *α*-glucosidase at a fourfold higher level than the positive control (acarbose), in a concentration-dependent manner. Moreover, blood glucose concentration was suppressed by SEMA* in vivo*. Clinical signs and weight changes were observed when conducting an evaluation of the acute toxicity of SEMA through a single-time administration, with clinical observation conducted more than once each day. After administration of the SEMA, observation was for 14 days; all of the animals did not die and did not show any abnormal symptoms. In addition, the inhibitory effects of rice coated with SEMA were evaluated in a group of impaired glucose tolerance patients on postprandial glucose and a group of normal persons, and results showed that SEMA had a clear inhibitory effect on postprandial hyperglycemia in both groups. Overall, SEMA showed excellent potential in the present study as a material for improving postprandial hyperglycemia.

## 1. Introduction

Diabetes mellitus is a metabolic disorder characterized by hyperglycemia caused by either abnormal control of insulin secretion in pancreatic cells or the deterioration of the action of insulin in peripheral tissues [1]. In particular, hyperglycemia after meals is induced by diastatic enzymes such as several proteins including hemoglobin, and it is a key factor in the etiology of many chronic diabetic complications including microvascular complications. The current classes of hypoglycemic agents used for the treatment of diabetes include insulin secretagogue, liver gluconeogenesis inhibitor, insulin sensitizer, and *α*-glucosidase inhibitor. The latter delays glucose absorption in the alimentary canal, and is widely used to lower postprandial glucose [2].

Intestinal *α*-glucosidase is an enzyme located in the microvillus membrane of the tunica mucosa and is essential for the digestion of carbohydrates. It breaks down polysaccharides into monosaccharides; for example, starch consumed via a meal is broken down into oligosaccharides by *α*-amylase and further metabolized into glucose by *α*-glucosidase [3]. *α*-Glucosidase inhibitors decrease postprandial glucose and effectively compensate for the insufficient insulin secretion in noninsulin dependent diabetes mellitus by combining with the enzyme and disturbing the dissolution and absorption of monosaccharides (i.e., glucose). While the *α*-glucosidase inhibitors currently on the market (acarbose and voglibose) advantageously do not induce hyperinsulinemia or hypoglycemia, it has been reported that their long-term use can cause side effects such as abdominal distention, vomiting, and diarrhea [4].

Recently, substitutional foods and natural products have been attracting attention owing to their potential for effective inhibitory action on postprandial hyperglycemia with decreased side effects. Many researchers are actively conducting studies to develop a hypoglycemic agent from natural products and food resources [5–7]. With a solution to the opening of imports in the rice market and changes in consumer perceptions about functional foods, their interest in functional coated rice, where bioactive substances are added gradually to the food, has increased; thus, to improve the quality of life of diabetic patients, the development of functional rice coated with antiglycemic agent has been attempted.

Intake of high glycemic index (GI) foods is restricted in diabetic patients with high blood glucose, owing to concern about the rapid rise of postprandial glucose. Developing a grain that does not increase blood glucose rapidly, and maintains taste, would have a profound impact on the quality of life of diabetic patients and quasi-healthy individuals (individuals with fasting blood glucose disorder, impaired glucose tolerance, obesity, and hyperlipidemia) and others who are concerned about their blood glucose.

*Morus alba* is a deciduous plant belonging to the family Moraceae. Its leaves contain flavonoids, steroids, triterpenes, amino acids, vitamins, and large quantities of minerals. In particular, the N-containing sugar deoxynojirimycin (DNJ) has been separated and identified from the* M. alba* leaf [8]. As a traditional herbal medicine, it is used to prevent and treat diabetes as well as quench thirst [6]. The water-soluble fractions of the* M. alba* leaf have been demonstrated to suppress blood glucose levels, via *α*-glucohydrolase suppression and the reduction of blood lipid concentrations [9, 10]. Several biological activities including the antioxidant activity of these water-soluble extracts and animal model have been reported [11, 12]. In addition,* Allium cepa* (onion) is a liliaceous biennial plant, which is known to have important biological activities such as the reduction of blood cholesterol, and effects on hypertension and diabetes [13–16].

This study aims to investigate the inhibitory activity of a standardized extract of* M. alba* leaf 50% ethanol extract (SEMA) on *α*-glucosidase, blood glucose reduction in both a normal animal model and a streptozotocin- (STZ-) induced diabetes model, and the acute toxicity, as well as evaluate its postprandial antihyperglycemic effect in impaired glucose tolerance patients and healthy individuals with cooked coated rice (CCR) contained SEMA.

## 2. Materials and Methods

### 2.1. Chemicals

**Figure 1:**
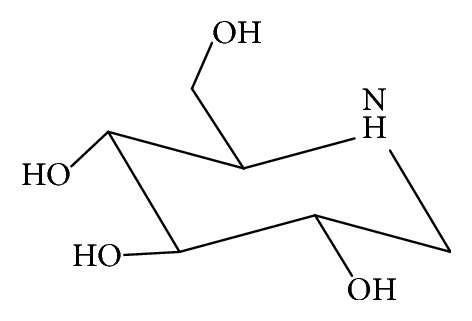
Chemical structure of 1-deoxynojirimycin (DNJ).

1-Deoxynojirimycin (DNJ, Figure 1), acetonitrile, glacial acetic acid (Merck, Germany), paraformaldehyde, ammonium acetate,* p*-nitrophenyl-*α*-D-glucopyranoside, STZ, soluble starch, urethane (Sigma Co., USA), and *α*-glucosidase (Wako, Japan) were purchased from their respective suppliers.

### 2.2. Preparation of a Standardized Extract of* M. alba* Leaf 50% Ethanol Extract

**Figure 2:**
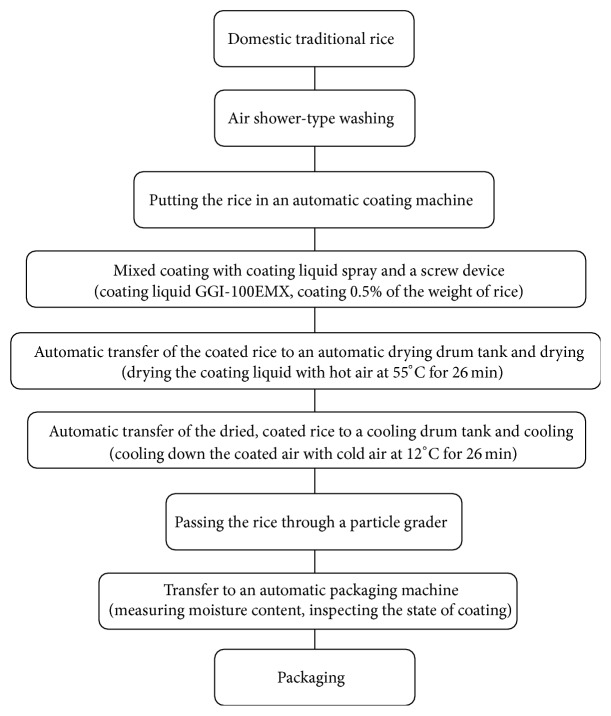
Coating process of standardized extract of* M. alba*.

SEMA was produced by mixing* M. alba* leaf 50% ethanol extract, water extract of onion, deep sea water, and sodium alginate with ethanol at ratios of 20.0, 58.6, 20.0, 0.5, and 0.9%. The coated rice was produced through a process of coating, drying, and cooling the complex extract after washing the plain rice (Figure 2).

### 2.3. Quantification of DNJ in a Standardized Extract of* M. alba* and Stability of Coated Rice after Heat Treatment (Cooking)

The DNJ content of the SEMA was measured with a method by Kimura et al. with some modification [17]. The supernatant was obtained via ultrasonic extraction (30 min) and centrifugation (7500 rpm, 30 min) with 50% acetonitrile (pH 5.5). 6.5 mM ammonium acetate as an extractant was used as a specimen of an analysis and the DNJ content, a functional component in rice coated with SEMA changed (stability) in the process of cooking, was investigated. A 300 mL solution of 50% acetonitrile (pH 5.5) and ammonium acetate (6.5 mM) was mixed with the coated rice, and ultrasonic extraction was conducted for 30 min. After nitrogen enrichment, the total volume was adjusted to 100 mL. After centrifugal separation (7500 rpm, 30 min), the supernatant was used as a specimen of analysis. To test the component concentrations after cooking, the rice (500 g) was first cooked using an electric rice cooker with distilled water (1500 mL); considering the weight ratios before and after cooking, the calculation was made by a conversion into the ratio of DNJ content to the weight of the rice. The extraction method was the same as that for the rice. A quantitative analysis of DNJ content was conducted using HPLC. The HPLC equipment consisted of the Agilent 1100 series with the TSK gel Amide-80 column (4.6 × 250 mm, 5 *μ*m, Tosoh bioscience, Japan) as the column of analysis. For the mobile phase, 80% acetonitrile (pH 5.5) containing ammonium acetate (6.5 mM) was used, which was analyzed using the isocratic method at a flow rate of 1.0 mL/min and injection volume of 10 *μ*L. DNJ was detected using an Evaporative Light Scattering Detector (ELSD, Alltech 2000 ES, USA), and analysis conditions were set up as follows: N_2_ gas pressure gauge, 2 kgf/cm^2^; drift tube heater, 105°C; nebulizer zone heater, 105°C; and gas flow, 2.0 L/min (19.0 psi).

### 2.4. Assay for the *α*-Glucosidase Inhibitory Activity

*α*-Glucosidase inhibitory activity was measured using a method from Kim et al. with some modification [18].* p*-Nitrophenyl-*α*-D-glucopyranoside was used as a substrate, with *α*-glucosidase as the enzyme. Change in absorbance of a control group (solvent only) and a processing group (specimen extract at a certain concentration) was observed to evaluate the degree of enzyme inhibition. BSA (bovine serum albumin) and NaN_3_ were dissolved in a PBS buffer, and the *α*-glucosidase enzyme (0.03 U/mL) was dissolved to make an enzyme solution.* p*-Nitrophenyl-*α*-D-glucopyranoside was dissolved in PBS at a concentration of 1 mM to make the substrate solution. Enzyme solution (60 *μ*L) was mixed with specimen extract (10 *μ*L) and water (130 *μ*L) was added. The absorbance of the specimen was measured at 405 nm. The same quantity of the enzyme solution and the specimen extract was then combined, and water (100 *μ*L) and substrate solution (30 *μ*L) were added before measuring absorbance at 405 nm for 10 min. The level of enzyme inhibitory activity was calculated using the following expression:

(1) Level  of  enzyme  inhibitory  activity%=1−SC×100,

where *S* is change of absorbance after specimen extract was added and *C* is change of absorbance in the control group in which no specimen was added.

### 2.5. Experimental Animals

Male Sprague-Dawley rats (180–200 g) were received from Koatech. Co., Ltd. and six-week-old male and female ICR mice were received from Central Lab Animal, Inc.; they were adapted to the feeding environment for one week before being used in diabetes and toxicity experiment, respectively. The feeding environment was maintained as follows: temperature, 23 ± 1°C; humidity, 60 ± 5%; noise, below 60 phones; air particulates, below 20 ppm; lighting, 150–300 lux; lighting time, 12 hours light/dark cycle; feed (Purina, Korea) and water,* ad libitum*. Breeding management of the animal experiments was carried out based on the “Guide for the Care and Use of Laboratory Animals,” and, after approval by the Ethics for Experiment Animals Committee of Hallym University, the animal experiments were conducted as follows.

#### 2.5.1. Diabetic Animal Model and Measuring Blood Glucose Reduction Effect

The experimental animals were fasted for 16 hours. STZ (0.1 M citrate buffer, 60 mg/kg) was administered intra-abdominally to induce diabetes. After fasting them for one week after STZ administration, blood glucose was measured from the tail veins using a blood glucose monitoring device (Accu-Chek, Germany). Animals that had fasting blood glucose over 250 mg/dL were considered diabetic and thus used in the experiments. Ten diabetic animals were randomly divided into three experimental groups: control, SEMA, and acarbose administration group. SEMA (480 mg/kg) and acarbose (20 mg/kg) were orally administered to their respective group once a day for 21 days. After fasting them on the last day of treatment, fasting blood glucose was measured, and soluble starch (2 g/kg) was administered 30 min after a meal. Blood glucose was measured from the tail vein at 30, 60, and 120 min after starch administration.

#### 2.5.2. Blood Biomarkers

After the experiments, blood was collected from the ophthalmic vein and placed in an SST vacutainer. Plasma was obtained by a centrifugal process at 3000 rpm for 15 min. Total protein, albumin, aspartate aminotransferase (AST), alanine aminotransferase (ALT), and alkaline phosphatase (ALP) showing the liver function index, and creatine showing the renal function index, were measured using a blood chemical measuring instrument (Thermo, USA).

#### 2.5.3. Acute Oral Toxicity

To evaluate toxicity, male and female ICR mice were divided into six groups (five male and five female mice for each group, resp.): a control and 5 different concentrations of SEMA suspended in 1% carboxymethyl cellulose. The volumes of oral administration were 1000, 500, 250, and 125 mg/kg, as well as 2000 mg/kg, the maximum value for the acute toxicity test. Weight change and survival were observed for 14 days [19].

### 2.6. Subjects

**Figure 3:**
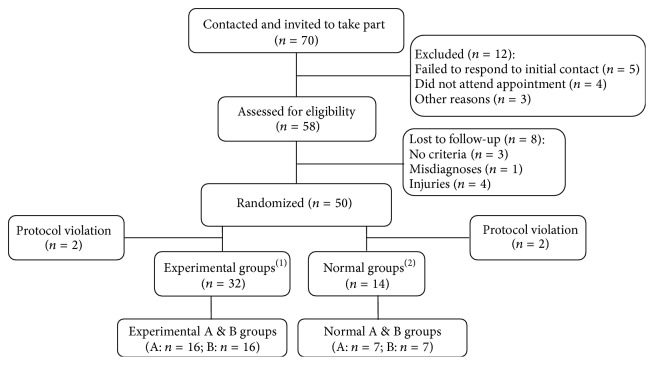
Flow of participants through each stage of the randomized trial. ^(1)^Experimental groups: impaired glucose tolerance patients (IGTP, females of 50–70 years); Exp. A: standardized extract of* M. alba *intake group, patients with impaired fasting glucose (*n* = 16); Exp. B: noncoated rice intake group, patients with impaired fasting glucose (*n* = 16). ^(2)^Normal groups: control group of experiment (14 healthy adults: 5 males and 9 females of 22–45 years); Nor. A: standardized extract of* M. alba *intake group-control (*n* = 7); Nor. B: noncoated rice intake group-control (*n* = 7).

Subjects diagnosed according to American Diabetes Association (ADA) criteria were selected among seventy impaired glucose tolerance patients (IGTP) and enrolled in this study [20, 21]. To measure the postprandial antihyperglycemic effect of cooked rice coated with SEMA, IGTP between females of 50–70 years were recruited with the criteria for selection as follows: impaired fasting blood between 100 and 125 mg/dL, weight over 50 kg and within ±30% of ideal body weight, understanding of the intention and content of the research, willingness to participate as subjects, and agreement to follow experimental procure. The flow diagram of the progress is shown in Figure 3. Fifty-three IGTP females and seventeen healthy adult subjects entered the study. Among them, forty-six (65.71%) completed the study and twenty-four individuals (34.29%) dropped out for reasons unrelated to the intervention like injuries, misdiagnoses, personal reasons, protocol violation, criteria, and other reasons. Criteria for exclusion included (1) patients who have or had a history of cancer of the liver, kidney, endocrine system, or blood, mental illness, or cardiovascular disease (angina pectoris, myocardial infarction, or cerebral infarction); (2) patients with a gastrointestinal disorder such as gastric atony or malabsorption syndrome or a chronic intestinal tract disease; (3) patients who had serum AST and ALT over 1.25 times higher than normal; (4) patients with blood creatine 1.5 mg/dL or higher; (5) patients with systolic blood pressure below 90 mmHg or over 160 mmHg or diastolic blood pressure under 60 mmHg or over 110 mmHg; (6) patients who took specialty pharmaceuticals (diabetes medications, insulin, steroid preparations, dopamine, dobutamine, female hormone therapy) or other oriental medicines that might affect blood glucose, within the last two weeks; (7) patients who were judged by the doctor in charge to be inappropriate for this experiment. Subjects were recruited at Hallym University Hospital and by a newspaper advertisement. In addition, fourteen healthy adults (five males and nine females, ages 22–45) were included as the subjects of the experiment as a control group to compare efficacy and this study consisted of one baseline visit and a visit at 3 months after randomization. At baseline, informed consent was obtained, and also demographic and treatment information was collected.

#### 2.6.1. Study Design

A glucose tolerance test was performed on the fourteen healthy adults (normal groups) and IGTP (experiment groups) after obtaining approval from the Institutional Review Board (IRB)/Ethics Committee of Hallym University Hospital. Subjects were randomized using a list prepared with a random number generator. The subjects in the control group were divided randomly into normal groups A and B (*n* = 7 for each group), as were the 32 persons in IGTP group (experiment groups A and B, *n* = 16 for each group). Normal group A (Nor. A) and experiment group A (Exp. A) were supplied with cooked rice coated of the SEMA, and normal group B (Nor. B) and experiment group B (Exp. B) were supplied with cooked rice without SEMA. In addition, all persons were supplied an equal amount of 75 g, respectively, of rice coated with SEMA and plain rice which were not different in color and taste. Subjects were limited to one locale until the completion of the glucose tolerance test. Light body motions were allowed, and reading materials, a computer, and films were provided. Taking in foods other than the meals provided was prohibited.

#### 2.6.2. Oral Glucose Tolerance Test

Capillary bloods from finger-pricks was collected from all subjects from 8 to 9 a.m., 30 min before a meal after fasting for over 12 hours, and 30 min, 1 h, and 2 h after a meal and three capillary blood samples were taken and an average of three readings was recorded. Blood glucose concentrations were measured using a simple blood glucose meter (Accu-Chek Go, Roche Diagnostics GmbH, Germany), 2 times during breakfast and lunch.

### 2.7. Statistical Analysis

Data are expressed as mean values ± SD and comparisons among data were carried out using Student's unpaired *t*-tests or one-way analyses of variance, as appropriate. Mean values were considered significantly different when *P* < 0.05.

## 3. Results and Discussion

### 3.1. Quantification of DNJ Content in SEMA and the Stability of Coated Rice after Heat Treatment (Cooking)

**Figure 4:**
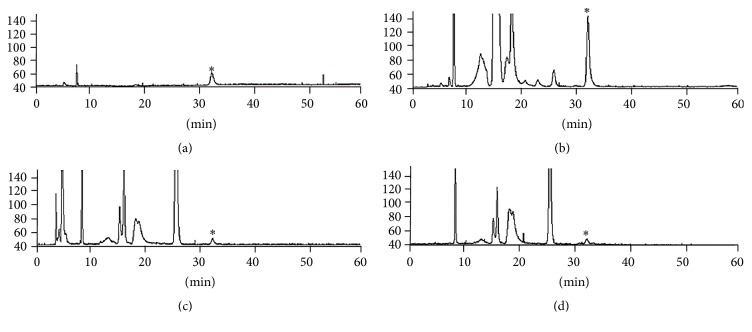
Chromatograms of standardized extract of* M. alba *and the extract of raw coated rice and cooked coated rice. (a) 1-Deoxynojirimycin (DNJ)^*∗*^, (b) standardized extract of* M. alba*, (c) raw coated rice, and (d) cooked coated rice.

In order to quantify the DNJ contained in SEMA, HPLC-ELSD analysis was used. As shown in Table 1, roughly 5.20 mg DNJ is contained in 1 g of SEMA. DNJ, a known hypoglycemic of the several bioactive substances contained in* M. alba* leaf, is a representative *α*-glucosidase inhibitor that inhibits the enzymatic reactions of *α*-glucosidase and mannose dehydrogenase, which decompose the disaccharides maltose, sucrose, and lactose into glucose [22]. In order to verify the stability of DNJ after cooking, HPLC-ELSD analysis was used. With the DNJ standard preparation, a standard calibration curve of peak area value obtained at each concentration was drawn, and, as a result, a regression equation, *y* = 1020*x* − 73.16, and correlation coefficient *R* = 0.999 were obtained. As shown in Table 1, raw coated rice (RCR) contained 12.27 mg DNJ per 100 g rice, while cooked coated rice (CCR) contained 11.7 mg DNJ per 100 g rice. DNJ content tended to decrease slightly after cooking, but there was no statistically significant difference. DNJ in the functional rice coated with SEMA did not undergo any significant changes after cooking (Figure 4).

### 3.2. Inhibitory Effect of SEMA on *α*-Glucosidase

An* in vitro* investigation of the *α*-glucosidase inhibitory activity of SEMA revealed that it inhibited 83.14% of *α*-glucosidase activity at a concentration of 0.8 mg/mL, while acarbose used as a positive control inhibited activity at a somewhat lower rate (69.38%). As shown in Table 2, while SEMA and acarbose both inhibited *α*-glucosidase activity in a concentration-dependent manner, SEMA showed about 4.5 times higher inhibitory activity (IC_50_) at 91.63 and 402.33 *μ*g/mL. SEMA is a preparation containing the highest ratio of* M. alba* leaf 50% ethanol extract, and it is suggested that its high *α*-glucosidase inhibitory activity is attributable to DNJ.

### 3.3. Effect of SEMA on Blood Glucose

**Figure 5:**
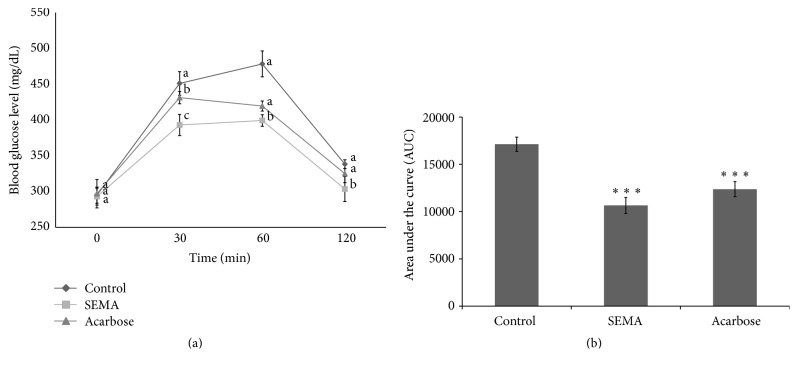
Changes in blood glucose levels in STZ-induced diabetic rats (a). Area under the blood glucose concentration curve was measured over 120 min (b) (AUC, 120 min). Values are expressed as the mean ± SE (*n* = 10). Different letters in the same time indicate significant differences, *P* < 0.05. ^*∗∗∗*^ *P* < 0.001 versus Con. SEMA (480 mg/kg) and acarbose (20 mg/kg) were orally administered to their respective group once a day for 21 days.

A considerable number of beta cells in the pancreas necrotize owing to the cytotoxic insult by STZ, and it is by the subsequent insulin depletion that hyperglycemia and diabetic phenomena are induced. A small number of beta cells in the pancreatic islet show excessive insulin secretion (early phase) in response to the hyperglycemic environment, but cell proliferation and differentiation are inhibited and the beta cells are exhausted by continued glucose-stimulated insulin secretion. An irreversible deficit of beta cells is caused in the pancreatic islets by this secondary injury caused by glucose toxicity [23, 24]. Thus, adjusting the blood glucose concentration is an important challenge for maintaining beta cell viability and preventing the progression of diabetes. In the diabetes model induced by STZ, changes in blood glucose after SEMA and starch administration were observed. The body weight of the rats tended to decrease rapidly after administration of STZ in all three groups (data not shown). It is hypothesized that the weight decreased as energy metabolism imbalance was induced by the destruction of pancreatic tissues by STZ administration. As shown in Figure 5(a), if SEMA was administered with starch in the diabetes model, at 60 min when the blood glucose of the experimental animals reached its maximum value, blood glucose was maintained at a lower level as compared to that of the control group (478.3 mg/dL). In other words, in the STZ-diabetic animal model after the administration of starch, blood glucose rapidly increased to 451.0 and 478.3 mg/dL at 30 and 60 min, respectively, in the control group, while the SEMA administration group maintained a significantly lower level of blood glucose at 393.0 and 399.3 mg/dL (*P* < 0.05) over the same time period. Ultimately, the SEMA administration group showed an increase in blood glucose from 451.0 to 478.3 mg/dL and 393.0 to 399.3 mg/dL at 30 and 60 min, respectively. As compared to the control group, there was a blood glucose reduction of 20.1% and 28.8% at 30 and 60 min after starch administration. Acarbose showed changes in the blood glucose of 431.2 and 419.5 mg/dL at 30 and 60 min, respectively, and there was a marked effect on blood glucose reduction, but no significant difference from the control group was observed (*P* < 0.05). When the AUC was compared between groups, the SEMA group significantly showed 27.74% reduction (*P* < 0.001) compared to the control group (Figure 5(b)).

### 3.4. Effect of SEMA on Blood Biomarkers

The results of a hematological analysis of total protein, albumin, AST, ALT, and creatine are shown in Table 3. There was significance in the total protein content; it was higher in the SEMA and acarbose administration groups than in the control group (*P* < 0.001). The albumin concentration followed the total protein results, with no significant difference between the experimental groups. Ju et al. noted that the plasma protein concentration is not easily affected by insulin and reported that the level of plasma protein in a normal rat is similar to that in a diabetic rat [25]. The level of serum AST activity significantly tended to decrease by 30.52% (*P* < 0.001) and 12.64% (*P* < 0.01) and the level of serum ALT activity tended to significantly decrease by 28.95 (*P* < 0.001) and 9.30% (*P* < 0.05) in the SEMA and acarbose administration groups, respectively, as compared to the control group, and statistical significance was confirmed. AST and ALT are discharged out of the cells after hepatocyte damage, increasing their concentration in the blood; hence, they are used as an index of liver injury; the AST and ALT activity increased with STZ administration in the present study and decreased after SEMA administration. The blood creatine concentration was high, but no significant difference was observed. Creatine is measured as indicator reflecting the status of renal function prior to diabetic nephropathy, with creatine being a substance that is generated as a result of the creatine phosphate metabolism in the muscle. Under normal conditions, creatine is isolated from the muscle at a relatively constant speed, so the blood creatine concentration is constant when filtered through the glomerulus and is neither reabsorbed nor metabolized [26]. In a chronic kidney disease due to diabetes, the glomerular filtration rate decreases and the concentration of blood creatine increases; in the present study, the creatine concentration tended to increase in the diabetes-induced group, but there was no significant difference. The degradation of kidney function can be assumed, but no serious complications appeared.

### 3.5. Effect of SEMA on Acute Oral Toxicity

The LD_50_ value is shown in Table 4. Clinical signs and weight change were observed by conducting an evaluation of the acute toxicity of SEMA through a single-time administration. After administration, rats were observed frequently on the first day, with clinical observation conducted more than once each day. No rats died or demonstrated any specific abnormal symptom over observation for 14 days. At all administration concentrations, no weight change had significance as compared to the control group (CMC), but a certain increase in weight was confirmed. Thus, LD_50_ of standardized extract of* M. alba* was demonstrated to be over 2 g/kg, and, within this range, no toxicity was observed. Throughout the present study, it was found that glycemic control by SEMA after a meal was caused by suppression of carbohydrate absorption by inhibiting *α*-glucosidase activity, and no toxicity of the extract was observed.

### 3.6. Impact of SEMA on Changes in Postprandial Glucose in Patients with Impaired Glucose Tolerance

**Figure 6:**
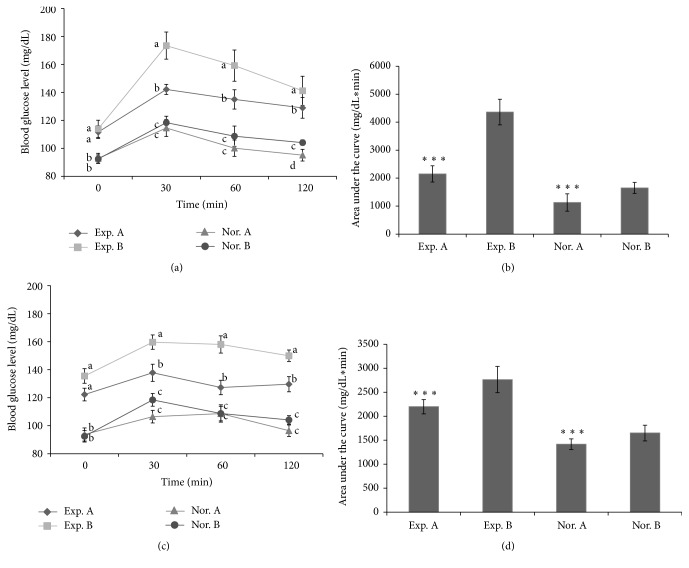
Changes in blood glucose levels after breakfast (a) and lunch (c). Area under the blood glucose concentration curve was measured over 120 min after breakfast (b) and lunch (d) (AUC, 120 min). Exp. A: standardized extract of* M. alba *intake group, patients with impaired fasting glucose. Exp. B: noncoated rice intake group, patients with impaired fasting glucose. Nor. A: standardized extract of* M. alba *intake group-control. Nor. B: noncoated rice intake group-control. Different letters in the same time indicate significant differences, *P* < 0.05. ^*∗∗∗*^ *P* < 0.001 versus Exp. B and Nor. B, respective group.

The seventy subjects were contacted and invited to take part in the trial. A total of seventy subjects attended clinic, five failed to respond to initial contact efforts, four did not attend the appointment, and three have other reasons. Twelve subjects were excluded: three subjects did not meet inclusion criteria, four participants were excluded due to protocol violation, and five subjects were excluded because of injuries and misdiagnoses. Forty-six cases successfully completed the trial (Figure 3). Changes in blood glucose before and after breakfast in the subjects are shown in Figure 6(a). There was no difference in the morning fasting glucose levels in the subjects of normal groups and experiment groups, but the level of blood glucose at 30 min after a meal of rice coated with SEMA (Exp. A) and cooked rice without SEMA (Exp. B) was 142.17 and 173.47 mg/dL, respectively; the blood glucose levels were significantly decreased (*P* < 0.05) in Exp. A in comparison to Exp. B (Figure 6(a)). This difference was maintained 1 or 2 h after a meal. In addition, the fasting glucose level was similar in normal groups, but the level of blood glucose at 30 min after a meal was not significantly about 11 mg/dL lower in Nor. A. When the AUC was compared between groups, Exp. A showed 49.28% reductions, respectively (*P* < 0.001), compared to Exp. B (Figure 6(b)). In the rice coated with SEMA, it was observed that there was a phenomenon in which the rapid rise in blood sugar after meals is effectively controlled. The above method was applied to lunch in the same way, and changes in blood glucose are shown in Figure 6(c). When blood glucose measured 30 min before a meal was compared between Exp. A and B, a high level of blood glucose was maintained at 13 mg/dL after breakfast in the Exp. B. Comparison between Exp. A and Exp. B 1 h and 2 h after lunch showed significantly decreased glucose of about 22 mg/dL in Exp. A after 30 min (*P* < 0.05), and this was similar to that observed 30 min after breakfast. It was observed that, in control groups, a glucose reduction effect of about 12 mg/dL was observed 30 min after lunch in group A that took rice coated with SEMA, while there was no difference between the groups 1 h or 2 h after lunch. While this was a randomized double-blind test, it is judged that SEMA has a clear effect on reducing postprandial glucose.

*M. alba* leaves are materials that have been widely used for diabetes and hyperglycemic control. This study found the antidiabetic effectiveness of SEMA. SEMA 1 g contains DNJ 5.20 mg and it showed about 4 times higher inhibitory activity against car *α*-glucosidase as compared to control medication acarbose. In STZ-diabetic animal model, blood glucose reduction effect was confirmed. In addition, in the acute toxicity evaluation, no toxicity was observed.* M. alba* extracts have been previously reported to have antihyperglycemic effect in STZ-induced diabetic rats [27] and in diet-induced obese mice [28] and to block inflammatory response in STZ-diabetic mice model [29] and in STZ-induced diabetic rats fed high-fat diet [30]. Among them, Jeszka-Skowron et al. have reported that DNJ may suggest additional roles in the gut to reduce or inhibit intestinal glucose digestion and absorption [30]. According to Kimura et al. analysis of the intake of healthy adults (average 25 years old) who were divided into four groups randomly showed that postprandial hyperglycemia was controlled more clearly in the group that took higher amount [31]. Moreover,* M. alba* leaves have been shown to exhibit antiglycation and antioxidant activity and postprandial hyperglycemia [32]. These results were shown to be similar to our antihyperglycemic effect.

In addition, it is very important for persons with glucose tolerance disability or diabetic patients to control their blood sugar so that postprandial glucose does not significantly increase [33]. For this reason, substances or foods effective for reduction of postprandial glucose can play an important role in the treatment of persons with glucose tolerance disability or diabetic patients. This study investigated the stability of SEMA and postprandial glucose reduction effect on patients with IGTP and a group of normal persons based on the results of *α*-glucosidase inhibitory activity of rice (grain) coatings (SEMA) made of standardized extracts and reduction of postprandial glucose in a model of STZ-induced diabetes. As a result, there was no change in the content of DNJ, a functional component of SEMA after cooking, so its stability was confirmed. In addition, in an evaluation of inhibitory effects of SEMA on reduction of postprandial glucose in a group of IGTP and a group of normal persons, there was a clear inhibitory effect of SEMA on postprandial hyperglycemia in both the group of IGTP and the group of normal persons that took SEMA. However, our results have been affected by small number of participants due to participants dropping out. Therefore, our results need more clinical trials which evaluate the diabetic patients including various types (ages, symptoms, and many diabetic patients) to confirm long-term efficacy and safety of* M. alba* in diabetic patients.

## 4. Conclusion

In summary, considering the present study and previous data, the results suggest that, at least in part,* M. alba* can be used as a nutraceutical agent to ameliorate hyperglycemic effect and can be used as an dietary supplementation for treatment of diabetic patients. Therefore, this study found that the plant extracts SEMA developed as a material for glycemic control have an excellent potential as a material improving postprandial hyperglycemia.

## Figures and Tables

**Table 1:** 1-Deoxynojirimycin concentration of a standardized extract of *M. alba*, raw coated rice, and cooked coated rice.

Samples	Concentration (DNJ contents)
Standardized extract of *M. alba*	5.20 ± 0.34^(1)^
Raw coated rice	12.27 ± 1.67^(2)^
Cooked coated rice	11.77 ± 1.67^(2)^

^(1)^mg/g standardized extract of *M. alba.*

^(2)^mg/100 g rice.

**Table 2:** Inhibitory effect of a standardized extract of *M. alba *on *α*-glucosidase activity.

Samples	IC_50_ (*μ*g/mL)
Standardized extract of *M. alba*	91.63 ± 0.92
Acarbose	402.33 ± 1.5

IC_50_ value is the concentration of sample required for 50% inhibition. Each value is expressed as mean ± SD in triplicate experiments.

**Table 3:** Effect of a standardized extract of *M. alba *on biochemical parameters.

	Control	Standardized extract of *M. alba*	Acarbose
Albumin	2.66 ± 0.05	2.80 ± 0.02	2.98 ± 0.18^*∗∗*^
Total protein	4.93 ± 0.12	5.59 ± 0.17^*∗∗∗*^	5.68 ± 0.22^*∗∗∗*^
AST^(1)^	133.6 ± 5.90	93.82 ± 4.03^*∗∗∗*^	116.7 ± 2.95^*∗∗*^
ALT^(2)^	91.40 ± 2.1	64.94 ± 7.34^*∗∗∗*^	82.9 ± 2.60^*∗*^
Creatine	0.55 ± 0.03	0.62 ± 0.01	0.63 ± 0.04

^(1)^AST: aspartate aminotransferase.

^(2)^ALT: alanine aminotransferase.

Data represent the mean ± SE (*n* = 10). Statistical significance of differences was calculated between control and experiment groups: ^*∗*^ *P* < 0.05, ^*∗∗*^ *P* < 0.01, and ^*∗∗∗*^ *P* < 0.001. SEMA (480 mg/kg) and acarbose (20 mg/kg) were orally administered to their respective groups once a day for 21 days.

**Table 4:** Mortality of male and female mice and body weight change after single oral administration of standardized extract of *M. alba *for 14 days (*n* = 5).

Sex	Dose (mg/kg)	Final mortality	LD_50_ (mg/kg)	Change of body weight (g)			
				Day after treatment			
				0	5	10	14
Male	0	0/5	>2,000	34.5 ± 1.7	37.5 ± 0.8	39.2 ± 0.9	40.8 ± 1.2
	125	0/5		34.3 ± 0.9	36.9 ± 1.5	38.3 ± 0.8	39.6 ± 1.1
	250	0/5		35.2 ± 0.4	37.9 ± 1.2	40.1 ± 1.1	41.9 ± 2.0
	500	0/5		34.5 ± 0.7	37.5 ± 0.8	34.5 ± 0.7	37.5 ± 0.8
	1,000	0/5		33.9 ± 0.9	36.9 ± 0.2	37.9 ± 0.9	39.8 ± 0.7
	2,000	0/5		34.6 ± 0.9	37.6 ± 2.0	39.9 ± 1.4	41.2 ± 1.6

Female	0	0/5	>2,000	31.8 ± 1.1	32.4 ± 1.0	33.2 ± 1.1	33.9 ± 1.2
	125	0/5		29.9 ± 0.9	31.9 ± 1.2	33.3 ± 1.1	33.8 ± 1.5
	250	0/5		30.1 ± 1.3	31.4 ± 1.4	32.4 ± 1.3	33.0 ± 1.5
	500	0/5		32.4 ± 0.9	33.1 ± 0.7	34.3 ± 0.9	34.9 ± 1.0
	1,000	0/5		29.3 ± 1.1	30.8 ± 1.2	31.6 ± 0.9	32.1 ± 1.0
	2,000	0/5		30.9 ± 1.1	31.2 ± 0.9	32.1 ± 1.3	32.9 ± 1.2

## References

[1] Moller D. E. (2001). New drug targets for type 2 diabetes and the metabolic syndrome. *Nature*.

[2] Krentz A. J., Bailey C. J. (2005). Oral antidiabetic agents: current role in type 2 diabetes mellitus. *Drugs*.

[3] Mooradian A. D., Thurman J. E. (1999). Drug therapy of postprandial hyperglycaemia. *Drugs*.

[4] Chiasson J.-L., Josse R. G., Hunt J. A. (1994). The efficacy of acarbose in the treatment of patients with non-insulin-dependent diabetes mellitus: a multicenter controlled clinical trial. *Annals of Internal Medicine*.

[5] Lee Y. R., Nam S. H., Kang M. Y. (2006). Hypoglycemic effect of the giant embryonic rice supplementation on streptozotocin-induced diabetic rats. *Korean Society of Food Science Technology*.

[6] Kim Y. Y., Cho R. W., Chung S. H., Koo S. J. (1999). Anti-hyperglycemic effect of cortex mori radicis in db/db mice. *Korean Society of Food Science Technology*.

[7] Ortiz-Andrade R. R., García-Jiménez S., Castillo-España P., Ramírez-Ávila G., Villalobos-Molina R., Estrada-Soto S. (2007). *α*-Glucosidase inhibitory activity of the methanolic extract from *Tournefortia hartwegiana*: an anti-hyperglycemic agent. *Journal of Ethnopharmacology*.

[8] Chen F., Nakashima N., Kimura I., Kimura M. (1995). Hypoglycemic activity and mechanisms of extracts from Mulberry leaves (Folium Mori) and Cortex Mori Radicis in streptozotocin-induced diabetic mice. *Journal of the Pharmaceutical Society of Japan*.

[9] Asano N., Tomioka E., Kizu H., Matsui K. (1994). Sugars with nitrogen in the ring isolated from the leaves of *Morus bombycis*. *Carbohydrate Research*.

[10] Nishioka T., Kawabata J., Aoyama Y. (1998). Baicalein, an *α*-glucosidase inhibitor from Scutellaria baicalensis. *Journal of Natural Products*.

[11] Lee J. S., Choi M. H., Jung S. H. (1995). Blood glucose-lowering effects of Mori folium. *Yakhak Hoeji*.

[12] Chen J., Li X. (2007). Hypolipidemic effect of flavonoids from mulberry leaves in triton WR-1339 induced hyperlipidemic mice. *Asia Pacific Journal of Clinical Nutrition*.

[13] Jain R. C., Vyas C. R. (1974). Hypoglycaemia action of onion on rabbits. *British Medical Journal*.

[14] Kawakishi S., Morimitsu Y. (1988). New inhibitor of platelet aggregation in onion oil. *The Lancet*.

[15] Makheja A. N., Vanderhoek J. Y., Bailey J. M. (1979). Effects of onion (Allium cepa) extract on platelet aggregation and thromboxane synthesis. *Prostaglandines and Medicine*.

[16] Weisenberger H., Grube H., Koenig E., Pelzer H. (1972). Isolation and identification of the platelet aggregation inhibitor present in the onion, *Allium cepa*. *FEBS Letters*.

[17] Kimura T., Nakagawa K., Saito Y. (2004). Determination of 1-deoxynojirimycin in mulberry leaves using hydrophilic interaction chromatography with evaporative light scattering detection. *Journal of Agricultural and Food Chemistry*.

[18] Kim Y.-M., Jeong Y.-K., Wang M.-H., Lee W.-Y., Rhee H.-I. (2005). Inhibitory effect of pine extract on *α*-glucosidase activity and postprandial hyperglycemia. *Nutrition*.

[19] Lee J. N., Park C. S., Kim H. P., Hwang S. Y., Chung W. G. (2002). Single dose toxicity study of Hwangjaegongjinbo, an invigorator, in mice and rats. *Journal of Toxicology and Public Health*.

[20] Hosseini S., Jamshidi L., Mehrzadi S. (2014). Effects of *Juglans regia* L. leaf extract on hyperglycemia and lipid profiles in type two diabetic patients: a randomized double-blind, placebo-controlled clinical trial. *Journal of Ethnopharmacology*.

[21] Rondanelli M., Opizzi A., Faliva M. (2014). Metabolic management in overweight subjects with naïve impaired fastin glycemia by means of a highly standardized extract from *Cynara scolymus*: a double-blind, placebo-controlled, randomized clinical trial. *Phytotherapy Research*.

[22] Asano N., Nishida M., Miyauchi M. (2000). Polyhydroxylated pyrrolidine and piperidine alkaloids from Adenophora triphylla var. japonica (Campanulaceae). *Phytochemistry*.

[23] Pipeleers D., Van De Winkel M. (1986). Pancreatic B cells possess defense mechanisms against cell-specific toxicity. *Proceedings of the National Academy of Sciences of the United States of America*.

[24] Garofano A., Czernichow P., Breant B. (2000). Impaired *β*-cell regeneration in perinatally malnourished rats: a study with STZ. *The FASEB Journal*.

[25] Ju J. S., Choi M., Koh E. S., Choi M. G. (1989). Effect of adrenal hormones and diets on diabetic rats. *Korean Journal of Nutrition*.

[26] Guyton A. C., Hall K. E. (1996). *Textbook of Medical Physiology*.

[27] Mohammadi J., Naik P. R. (2008). Evaluation of hypoglycemic effect of *Morus alba* in an animal model. *Indian Journal of Pharmacology*.

[28] Katsube T., Yamasaki M., Shiwaku K. (2010). Effect of flavonol glycoside in mulberry (*Morus alba* L.) leaf on glucose metabolism and oxidative stress in liver in diet-induced obese mice. *Journal of the Science of Food and Agriculture*.

[29] Guo C., Li R., Zheng N., Xu L., Liang T., He Q. (2013). Anti-diabetic effect of ramulus mori polysaccharides, isolated from Morus alba L., on STZ-diabetic mice through blocking inflammatory response and attenuating oxidative stress. *International Immunopharmacology*.

[30] Jeszka-Skowron M., Flaczyk E., Jeszka J., Krejpcio Z., Król E., Buchowski M. S. (2014). Mulberry leaf extract intake reduces hyperglycaemia in streptozotocin (STZ)-induced diabetic rats fed high-fat diet. *Journal of Functional Foods*.

[31] Naowaboot J., Pannangpetch P., Kukongviriyapan V., Kongyingyoes B., Kukongviriyapan U. (2009). Antihyperglycemic, antioxidant and antiglycation activities of mulberry leaf extract in streptozotocin-induced chronic diabetic rats. *Plant Foods for Human Nutrition*.

[32] Kimura T., Nakagawa K., Kubota H. (2007). Food-grade mulberry powder enriched with 1-deoxynojirimycin suppresses the elevation of postprandial blood glucose in humans. *Journal of Agricultural and Food Chemistry*.

[33] Jenkins D. J. A., Wolever T. M. S., Taylor R. H. (1981). Glycemic index of foods: a physiological basis for carbohydrate exchange. *The American Journal of Clinical Nutrition*.

